# Overstrained Brains : A Cry for More Institutions

**Published:** 1893-03-11

**Authors:** Walter J. Eccok

**Affiliations:** Lynn Road, Ely, Cambs


### HOSPITAL OPINION.

[Contributions critical, suggestive, controversial, and technical are invited for this column, which is open to everyone who has anything to say that is of practical value and interest.]

### OVERSTRAINED BRAINS: A CRY FOR MORE INSTITUTIONS.

Sir,—In the course of the last few years my attention has been drawn to a class of cases which seem to require special treatment, viz.: cases of mental breakdown from over-strain in preparation for examinations. Usually the age of the person so afflicted is from 18 to 21; and I think it is more often a girl than a youth.

At present there seems no option for parents of the middle-class (in which the cases are most frequent) but to send the patient to a lunatic asylum, public or private. If the patients recover and return to ordinary life, as in the majority of cases, there seems little reason why they should not, they bear the same stigma that they would if actually "harmless lunatics." It is a difficulty to find them employment of a congenial order, and they are more or less a drag on their parents for an indefinite period. In all likelihood the breakdown has occurred in preparing for some official employment and the fact of having passed through a lunatic asylum is a fatal and permanent bar to official life.

The question that has been put to me more than once is—considering the growing frequency of these cases—should not special homes for treatment of mental overstrain, as distinct from those of ordinary insanity, be established? It has been suggested to me that perhaps the Editor of The Hospital might devote a leader to the subject in an early number, and so initiate a small crusade in the general press. And in furtherance of that suggestion I have ventured to write you this letter, in the hope that you may see your way to lighten the heavy hearts of a good many parents on whose children misfortune of this kind has fallen.—I am, dear Sir, yours faithfully, Walter J. Eccok, B.A.Lond.

Lynn Road, Ely, Cambs.

